# Comparative Evaluation of Herbal and Conventional Endodontic Irrigants on Postoperative Pain and Substance P Levels in Patients With Symptomatic Irreversible Pulpitis and Apical Periodontitis

**DOI:** 10.7759/cureus.100669

**Published:** 2026-01-03

**Authors:** Lubna Hassan, Sonali Taneja, Vidhi Bhalla, Setu Katyal

**Affiliations:** 1 Department of Conservative Dentistry and Endodontics, Ghaziabad Dental Care, Ghaziabad, IND; 2 Department of Conservative Dentistry and Endodontics, I.T.S Centre for Dental Studies and Research, Ghaziabad, IND; 3 Department of Conservative Dentistry and Endodontics, Maulana Azad Institute of Dental Sciences, New Delhi, IND

**Keywords:** endodontics, herbal, irreversible pulpitis, irrigants, pain

## Abstract

Introduction

Postoperative pain is a common complication following root canal treatment (RCT) in teeth with symptomatic irreversible pulpitis and apical periodontitis. This study aimed to compare the effects of ketorolac tromethamine, 100% *Moringa oleifera* leaf extract, 50% *Azadirachta indica* leaf extract, and 5.25% sodium hypochlorite, as final root canal irrigants, on postoperative pain intensity and Substance P expression in patients with symptomatic irreversible pulpitis and apical periodontitis.

Materials and methods

In this prospective observational study, 84 patients with maxillary or mandibular molars diagnosed with symptomatic irreversible pulpitis and apical periodontitis were divided into four groups (21 patients each) based on the final irrigant used during single-visit RCT. Preoperative and postoperative Substance P levels were measured in pulp and periapical blood/exudate samples using enzyme-linked immunosorbent assay (ELISA). Pain intensity was assessed preoperatively and 24 hours postoperatively using a visual analog scale (VAS). Statistical analyses included paired t-tests, Wilcoxon signed-rank tests, one-way analysis of variance (ANOVA), Kruskal-Wallis tests, post-hoc comparisons, and Pearson correlation.

Results

All irrigants were associated with significant intragroup reductions in both Substance P levels and pain intensity at 24 hours (p = 0.001). Preoperative Substance P levels showed significant intergroup differences, which largely resolved postoperatively. Postoperative pain scores differed significantly between groups, with *A. indica* being associated with higher pain than the other three irrigants, which demonstrated comparable outcomes. No consistent correlation was found between Substance P levels and pain scores.

Conclusion

All tested irrigants effectively reduced postoperative pain and Substance P expression. Sodium hypochlorite, ketorolac tromethamine, and *M. oleifera* leaf extract provided similar analgesic efficacy, which was superior to that of *A. indica*. Alternative irrigants may serve as viable adjuncts in clinical practice, particularly when enhanced biocompatibility or anti-inflammatory effects are desired.

## Introduction

Endodontic treatment is essential for managing pulpal and periapical infections caused by diverse microorganisms in the root canal system. The success of root canal treatment (RCT) hinges on meticulous procedures, including biomechanical preparation, thorough disinfection, and effective obturation, which collectively aim to eradicate the microbial load and promote tissue healing [1]. However, postoperative pain remains a significant challenge, affecting 1.7% to 80% of patients, particularly within the first 24 hours post-treatment [2,3]. This pain arises from periapical inflammatory reactions triggered by mechanical, chemical, or microbial factors and is influenced by variables such as patient demographics (age and gender), tooth characteristics (vitality and canal number), and procedural elements (irrigation technique and number of visits) [2-4]. Inflammation and pain are intricately linked in endodontic pathology, involving microvascular changes in the pulp, plasma extravasation, and the release of mediators such as bradykinin, arachidonic acid metabolites, cytokines, and neuropeptides from sensory nerves and immune cells [5]. Notably, Substance P, a neuropeptide, amplifies inflammation and peripheral sensitization in pulpitis, warranting clinical evaluation during the treatment stages [6].

Effective infection control is paramount; however, mechanical instrumentation alone cannot eliminate all debris, underscoring the role of irrigants. These solutions must exhibit broad antimicrobial activity, tissue dissolution, endotoxin neutralization, and smear layer removal, while being biocompatible and non-irritant [7]. Sodium hypochlorite is widely used for its antimicrobial and tissue-dissolving properties; however, it can provoke periapical irritation at higher concentrations [8]. Amid the growing interest in natural alternatives, herbal products such as neem (*Azadirachta indica*) and *Moringa oleifera* offer biocompatibility, antimicrobial, anti-inflammatory, and antioxidant benefits, potentially mitigating pain and inflammation [9,10]. Additionally, ketorolac tromethamine, a potent non-steroidal anti-inflammatory drug (NSAID) that inhibits cyclooxygenase-1, has emerged as a candidate irrigant with anti-inflammatory potential [11]. Despite these prospects, comparative evidence on herbal irrigants versus sodium hypochlorite is scarce, highlighting the need for targeted research in this area.

This study aimed to investigate the efficacy of ketorolac tromethamine, 100% *M. oleifera* leaf extract, 50% *A. indica* leaf extract, and 5.25% sodium hypochlorite as root canal irrigants on the intensity of postoperative pain and Substance P expression in patients with symptomatic irreversible pulpitis and symptomatic apical periodontitis. The objectives of this study were to evaluate and compare the efficacy of these irrigants on postoperative pain intensity and Substance P expression in such patients.

## Materials and methods

Study design and setting

This prospective observational study was conducted in the Department of Conservative Dentistry and Endodontics at I.T.S. Centre for Dental Studies and Research, Ghaziabad, India, from August 2024 to August 2025. This study observed the effects of different root canal irrigants used in routine clinical practice on postoperative pain intensity. Patients were treated with irrigants selected based on standard clinical protocols and availability, without randomization or intervention assignments. The study was approved by the Institutional Ethical Committee (ITSCDSR/Director-Principal/2024/L/219) and registered in the Clinical Trials Registry of India (CTRI) (CTRI/2024/08/073074). All procedures followed ethical principles, and written informed consent was obtained from all participants.

Sample size and patient selection

The sample size was calculated using G*Power software (version 3.1.9.2; Heinrich Heine University, Düsseldorf, Germany). With an alpha error of 0.05, a power of 95%, and an effect size of 0.5 (derived from a previous study by Bamini et al. [12] to ensure reliable detection of a difference in Substance P levels), the analysis indicated a requirement of 72 patients (18 per group). To account for an anticipated 10% dropout rate in each group, the total sample size was increased to 84 patients (21 patients per group).

Patients aged 18-65 years, presenting with symptomatic irreversible pulpitis and apical periodontitis (periapical index (PAI) score 1-3) [13] in maxillary or mandibular molars, were included. The PAI index is a scoring system freely available with open access. The inclusion criteria comprised a prolonged positive response to cold test (Hygienic EndoIce, Coltene, Altstätten, Switzerland) and electric pulp tester (Parkell, Brentwood, NY, USA), moderate to severe preoperative pain (visual analog scale (VAS) 4-10) [14], and systemic health (American Society of Anesthesiologists physical status classification system Class I) [15]. The exclusion criteria were as follows: medically compromised patients, asymptomatic or non-vital teeth, advanced periodontal disease, open apex or resorption, allergy to materials, pregnancy, and recent analgesic or antibiotic use.

A total of 84 patients diagnosed with symptomatic irreversible pulpitis and apical periodontitis were included in the study and allocated into four groups, with 21 patients in each group, based on the final irrigant used during single-visit RCT. Group 1 received 5.25% sodium hypochlorite as the final irrigant, Group 2 received ketorolac tromethamine, Group 3 received 100% *M. oleifera* leaf extract, and Group 4 received 50% *A. indica* (neem) leaf extract. 

100% *M. oleifera* leaf extract

Fresh *M. oleifera* leaves were harvested by hand from mature trees, thoroughly washed under running tap water to remove debris, and drained. The leaves were shade-dried at room temperature for 72 hours until crisp, followed by oven drying at 40°C for 48 hours in a hot air oven (Memmert, Buechenbach, Germany) to ensure complete moisture removal. The dried leaves were then pulverized into a fine powder using a high-speed blender (Philips, Jakarta, Indonesia). To prepare the 100% extract, 100 g of powdered leaves were added to 100 mL of distilled water preheated to 90°C (±20°C). The mixture was allowed to steep for 30 minutes, with occasional stirring to facilitate decoction. Subsequently, the extract was filtered while hot through a Whatman filter paper under vacuum using a Büchner funnel. Additional hot distilled water was passed through the residue to achieve a final volume of 100 mL, yielding a concentrated 100% *M. oleifera* leaf extract. The extract was stored in sterile amber bottles at 4°C until use and warmed to room temperature prior to clinical application.

50% *A. indica* (neem) leaf extract

Fresh *A. indica* leaves were collected manually, cleaned under running tap water, and air-dried in the shade for five days to prevent the loss of active compounds. The dried leaves were oven-dried at 40°C for 24 hours (Memmert, Buechenbach, Germany) and ground into a fine powder using a food grinder (Philips, Jakarta, Indonesia). For the 50% extract, 50 g of powdered neem leaves were mixed with 100 mL of distilled water maintained at 90°C (±20°C). The decoction was prepared by boiling the mixture gently for 30 minutes, followed by hot filtration through a Büchner funnel with Whatman filter paper. The filtrate was adjusted to 100 mL by adding hot distilled water to the residue during the filtration. The resulting 50% neem leaf extract was aliquoted into sterile containers, refrigerated at 4°C, and brought to room temperature before irrigation. These herbal extracts were prepared weekly under aseptic conditions to maintain potency and sterility, ensuring consistency in bioactive compound concentration for clinical use.

Clinical procedures

Preoperative pain was assessed using a 10-point VAS [14]. The VAS scale is free to use for research purposes. Local anesthesia was achieved using 2% lidocaine with 1:100,000 epinephrine (Icpa Health Products, Mumbai, India). Tooth isolation was performed using a rubber dam (Coltene, Altstätten, Switzerland). Access cavity preparation involved sterile burs, and a pulp blood sample (S1) was collected using a sterile paper point. The working length was determined electronically (Dentaport ZX, J. Morita Corp., Osaka, Japan) and radiographically confirmed.

Biomechanical preparation was performed using ProTaper Gold rotary files (Dentsply-Tulsa, Charlotte, NC, USA) up to F2 in the crown-down technique, with 17% ethylenediaminetetraacetic acid (EDTA) (Waldent, New Delhi, India) for lubrication and smear layer removal. Intermediate irrigation employed 5.25% sodium hypochlorite (Calyx Chemicals and Pharmaceuticals Ltd., Maharashtra, India). The final irrigation (5 mL for one minute) used one of the following: 5.25% NaOCl, 100% *M. oleifera* leaf extract, ketorolac tromethamine (Dr. Reddy’s Laboratories, Hyderabad, India), or 50% *A. indica* leaf extract, delivered via a 30G side-vented needle (Dispovan, Hindustan Syringes & Medical Devices, Faridabad, India) with ultrasonic activation (Irrisafe tip, Satelec-Acteon, Mérignac, France).

A periapical blood sample (S2) was collected post-preparation using a sterile paper point placed 1-2 mm beyond the apex. The obturation was performed using gutta-percha with AH Plus sealer (Dentsply DeTrey, Konstanz, Germany) via lateral condensation. Occlusion was relieved, and a composite resin was used for restoration. Single-visit treatment was performed in this case.

Outcome assessment

Postoperative pain was evaluated at 24 hours using the VAS. Substance P levels in the S1 (preoperative pulp blood) and S2 (post-preparation periapical blood/exudate) samples were quantified using a commercial competitive enzyme-linked immunosorbent assay (ELISA) kit: the Substance P Parameter Assay Kit (Catalog No. KGE007; R&D Systems, Inc., Minneapolis, MN, USA, part of Bio-Techne). This kit was selected for its high sensitivity (minimum detectable dose ~43.8 pg/mL), specificity for multi-species Substance P (including human), and validation for biological fluids such as serum, plasma, saliva, and urine, making it suitable for small-volume dental samples. This study was conducted as a single-blind study. The patients were blinded to the type of final irrigant used during RCT, while the operator was aware of the irrigant being administered due to the nature of the clinical procedures. Outcome assessment for postoperative pain and laboratory analysis of Substance P levels were performed without disclosure of group allocation.

Statistical analysis

Data were entered into Microsoft Excel (Microsoft® Corp., Redmond, WA, USA) and subsequently analyzed using IBM SPSS Statistics for Windows, Version 23.0 (Released 2015; IBM Corp., Armonk, NY, USA). Results for Substance P levels and pain (VAS) were presented as median, mean, and standard deviation. For Substance P, intergroup comparisons were conducted using one-way analysis of variance (ANOVA), and intragroup comparisons across time intervals were conducted using paired t-tests. The Shapiro-Wilk test indicated that VAS data were not normally distributed; therefore, the non-parametric Kruskal-Wallis test was used for intergroup comparison of pain scores, and the Wilcoxon signed-rank test was used for intragroup comparisons across time. Pearson’s correlation analysis was conducted between Substance P levels and VAS scores. Statistical significance was set at p < 0.05.

## Results

The mean age across the groups ranged from approximately 34 to 38 years, with no statistically significant difference, as determined by a one-way ANOVA test (p = 0.587). Furthermore, the distribution of sex was nearly identical, with no significant variation found using the chi-square test (p = 0.986). This demographic equivalence between the groups supports the internal validity of the study, indicating that subsequent findings are unlikely to be biased by these fundamental patient characteristics (Table 1).

**Table 1:** Baseline demographic characteristics of participants in the study groups. Values are presented as mean ± standard deviation (SD) for age and number (%) for sex distribution. *One-way analysis of variance (ANOVA) test, **Chi-square test. p > 0.05 denotes no statistical significance. Group 1 (5.25% sodium hypochlorite), Group 2 (ketorolac tromethamine), Group 3 (100% *Moringa oeifera*), Group 4 (50% *Azadirachta indica)*.

Parameters	Category	Group 1	Group 2	Group 3	Group 4	Test value	p-value
Age	Years (mean ± SD)	37.76 ± 9.70	36.14 ± 8.85	35.10 ± 8.75	33.81 ± 10.41	0.65	0.587*
Sex	Male n (%)	10 (47.6%)	10 (47.6%)	10 (47.6%)	11 (52.4%)	0.14	0.986**
	Female n (%)	11 (52.4%)	11 (52.4%)	11 (52.4%)	10 (47.6%)		

Table 2 reveals a statistically significant reduction in Substance P levels from the preoperative to postoperative period within all four study groups, as determined by paired t-tests (p = 0.001 for each). Although the mean values consistently decreased, the magnitude of change, as indicated by the effect size, varied notably between the groups. Specifically, Group 4 demonstrated the largest effect (0.81), followed by Group 1 (0.69), whereas Groups 2 and 3 showed smaller negative effect sizes. This suggests that, while the intervention was associated with a significant decline in Substance P levels, its relative impact differed among the groups (Table 2).

**Table 2:** Intragroup comparison of preoperative and postoperative Substance P levels in picograms per milliliter (pg/mL) in the study groups. Values are presented as median and mean ± standard deviation (SD). Intragroup comparisons were performed using the paired t-test, effect size reported as Cohen’s d. *p = 0.001 denotes statistically significant values. Group 1 (5.25% sodium hypochlorite), Group 2 (ketorolac tromethamine), Group 3 (100% *Moringa Oeifera*), Group 4 (50% *Azadirachta Indica*).

Groups	Substance P (pg/mL) (preoperative)	Substance P (pg/mL) (postoperative)	T statistic	p-value	Effect size
Groups	Mean ± SD	Mean ± SD			
Group 1	19.71 ± 1.74	15.70 ± 1.83	13.02	0.001*	0.69
Group 2	20.43 ± 1.29	14.60 ± 1.77	11.01	0.001*	-0.24
Group 3	21.97 ± 1.46	15.82 ± 1.14	13.48	0.001*	-0.29
Group 4	19.25 ± 1.80	16.23 ± 1.88	11.87	0.001*	0.81

Table 3 indicates that there were statistically significant differences in Substance P levels between the study groups, both before and after the intervention. A one-way ANOVA revealed a significant difference in preoperative levels, with a strong effect size (0.44), suggesting notable baseline variability. Postoperatively, a significant intergroup difference remained (p = 0.017), but the effect size was considerably smaller (0.14). This reduction in effect size implied that the intervention moderated the initial disparities, leading to more homogenized Substance P levels across the groups after the procedure.

**Table 3:** Intergroup comparison of preoperative and postoperative Substance P levels in picograms per milliliter (pg/mL) among study groups. Values are presented as mean ± standard deviation. Intergroup comparisons were performed using one-way analysis of variance (ANOVA). *p < 0.05 is considered statistically significant.

Variable	Group 1 (5.25% sodium hypochlorite)	Group 2 (ketorolac tromethamine)	Group 3 (100% *Moringa oleifera*)	Group 4 (50% *Azadirachta indica*)	F statistic	p-value	Effect size
Substance P (pg/mL) (preoperative)	19.71 ± 1.74	20.43 ± 1.29	21.97 ± 1.46	19.25 ± 1.80	11.82	0.001*	0.44
Substance P (pg/mL) (postoperative)	15.70 ± 1.83	14.60 ± 1.77	15.82 ± 1.14	16.23 ± 1.88	3.61	0.017*	0.14

Table 4 details the specific pairwise differences between groups for Substance P levels, as identified by post-hoc Tukey’s test. Significant baseline differences were observed in the preoperative measurements. Group 3 had a substantially higher mean Substance P level than Groups 1, 2, and 4. However, this pattern changed dramatically postoperatively. The only statistically significant pairwise difference that remained was between Groups 2 and 4, where Group 4 maintained a higher level. All other postoperative comparisons, including those involving the initially high Group 3, were not significant. This shift indicates that, while the groups began with heterogeneous baseline levels, the intervention effectively reduced most intergroup disparities, leading to a more uniform postoperative state, with one exception.

**Table 4:** Post-hoc pairwise comparison of Substance P levels in picograms per milliliter (pg/mL) among study groups using Tukey’s test. Post-hoc analysis performed following one-way analysis of variance (ANOVA) using Tukey’s honestly significant difference (HSD) test, mean difference represents intergroup differences in Substance P levels. *p < 0.05 is considered statistically significant. Group 1: 5.25% sodium hypochlorite, Group 2: ketorolac tromethamine, Group 3: 100% *Moringa oleifera*, Group 4: 50% *Azadirachta indica*.

Pairwise comparison	Substance P (preoperative)			Substance P (postoperative)		
	Mean difference (pg/mL)	T statistic	p-value	Mean difference (pg/mL)	T statistic	p-value
Group 1 - Group 2	-0.71	-1.46	0.895	1.10	2.12	0.223
Group 1 - Group 3	-2.26	-4.61	0.001*	-0.13	-0.25	0.999
Group 1 - Group 4	0.47	0.95	0.999	-0.53	-1.02	0.999
Group 2 - Group 3	-1.54	-3.15	0.014*	-1.23	-2.37	0.123
Group 2 - Group 4	1.18	2.41	0.109	-1.63	-3.14	0.014*
Group 3 - Group 4	2.72	5.56	0.001*	-0.40	-0.78	0.999

Table 5 demonstrates a statistically significant reduction in postoperative pain, as measured by the VAS score, within each study group. The Wilcoxon paired test confirmed that the decline from the preoperative to postoperative period was highly significant for all four groups (p = 0.001 for each). This indicates that, although the intervention was effective in significantly lowering pain universally, the degree of analgesic efficacy, as reflected in the final VAS scores, was comparatively lower in Group 4.

**Table 5:** Intragroup comparison of preoperative and postoperative visual analog scale (VAS) pain scores in the study groups. Values are presented as median (range) and interquartile range (IQR). Intragroup comparisons were performed using the Wilcoxon signed-rank test. *p = 0.001 denotes statistically significant values. Group 1 (5.25% sodium hypochlorite), Group 2 (ketorolac tromethamine), Group 3 (100% *Moringa oeifera*), Group 4 (50% *Azadirachta indica*). Pain scores were evaluated using the VAS scale [14].

Wilcoxon paired test	VAS (preoperative)		VAS (postoperative)		Z statistic	p-value
	Median (IQR)	Rank	Median (IQR)	Rank		
Group 1	7 (2)	34.86	3 (1)	47.36	4.04	0.001*
Group 2	8 (2)	47.76	3 (1)	30.71	4.06	0.001*
Group 3	8 (1.5)	48.64	2 (1)	29.52	4.04	0.001*
Group 4	7 (1.5)	38.74)	4 (1.5)	62.41	4.06	0.001*

Table 6 shows the intergroup differences in pain scores (VAS) before and after the intervention. The Kruskal-Wallis test indicated no statistically significant difference in preoperative pain levels among the four study groups, confirming comparable baseline pain. However, a highly significant difference was observed during the postoperative period. This finding indicates that, while all groups started with similar pain, the interventions led to significantly different analgesic outcomes, with Group 3 showing the strongest, and Group 4 the weakest effect.

**Table 6:** Intergroup comparison of preoperative and postoperative visual analog scale (VAS) pain scores among study groups. Values are presented as median (mean rank). Intergroup comparisons were performed using the Kruskal-Wallis test. *p < 0.05 is considered statistically significant. Group 1: 5.25% sodium hypochlorite, Group 2: ketorolac tromethamine, Group 3: 100% *Moringa oleifera*, Group 4: 50% *Azadirachta indica*. Pain scores were evaluated using the VAS scale [14].

Parameter	Group 1		Group 2		Group 3		Group 4		H statistic	p-value
	Median (IQR)	Rank	Median (IQR)	Rank	Median (IQR)	Rank	Median (IQR)	Rank		
VAS (preoperative)	7 (2)	34.86	8 (2)	47.76	8 (1.5)	48.64	7 (1.5)	38.74	5.23	0.155
VAS (postoperative)	3 (1)	47.36	3 (1)	30.71	2 (1)	29.52	4 (1.5)	62.41	27.15	0.001*

Based on the post-hoc Bonferroni analysis in Table 7, postoperatively, Group 4 reported significantly higher pain than all other groups, compared to Groups 1, 2, and 3. Conversely, there were no significant postoperative differences among Groups 1, 2, and 3. This confirms that the significant overall intergroup difference (Table 6) is primarily driven by the inferior analgesic outcome in Group 4. Preoperatively, although the overall Kruskal-Wallis test was not significant (Table 6), some pairwise comparisons reached Bonferroni-adjusted significance (Group 1 vs Group 2 and Group 1 vs Group 3). These differences reflect minor rank-based variations and do not indicate a clinically meaningful baseline imbalance.

**Table 7:** Post-hoc pairwise comparison of visual analog scale (VAS) pain scores among study groups using Bonferroni correction. Post-hoc analysis performed following Kruskal-Wallis test using Bonferroni-adjusted pairwise comparisons, adjusted p-values reported. *p < 0.05 is considered statistically significant. Pain scores were evaluated using the VAS scale [14].

Pairwise comparison	VAS (postoperative)		
	Mean rank difference	H statistic	Adjusted p-value
Group 1 - Group 2	16.64	2.24	0.011*
Group 1 - Group 3	17.83	2.43	0.007*
Group 1 - Group 4	-15.05	2.05	0.010*
Group 2 - Group 3	1.19	0.16	0.430
Group 2 - Group 4	-31.69	4.33	0.001*
Group 3 - Group 4	-32.88	4.49	0.001*

Pearson’s correlation analysis revealed consistently weak associations between Substance P levels and VAS pain scores across all groups, both preoperatively and postoperatively. The r values ranged from -0.29 to 0.19, indicating a negligible linear relationship. The direction of the correlation was inconsistent; some groups showed slightly positive r values, while others showed slightly negative values. This pattern of low and variable r values demonstrates that changes in Substance P levels did not show a strong or consistent correlative relationship with reported pain intensity in this study cohort (Table 8).

**Table 8:** Pearson correlation analysis between Substance P levels and visual analog scale (VAS) pain scores in the study groups. Correlation analysis was performed using Pearson’s correlation coefficient (r), with the confidence interval (CI) indicating 95% confidence. p > 0.05 is considered not statistically significant. Pain scores were evaluated using the VAS scale [14].

Correlation	Substance P levels	VAS scores	r-value	p-value	CI at 95%
Group 1 (5.25% sodium hypochlorite)	Preoperative	Preoperative	-0.05	0.841	-0.47, 0.39
	Postoperative	Postoperative	-0.29	0.211	-0.64, 0.16
Group 2 (ketorolac tromethamine)	Preoperative	Preoperative	0.13	0.575	-0.32, 0.5
	Postoperative	Postoperative	0.18	0.447	-0.28, 0.56
Group 3 (100% *Moringa oleifera*)	Preoperative	Preoperative	0.19	0.416	-0.27, 0.57
	Postoperative	Postoperative	-0.26	0.255	-0.62, 0.19
Group 4 (50% *Azadirachta indica*)	Preoperative	Preoperative	0.11	0.634	-0.34, 0.52
	Postoperative	Postoperative	-0.18	0.427	-0.57, 0.27

## Discussion

The present study evaluated the effects of four root canal irrigants - 5.25% sodium hypochlorite, ketorolac tromethamine, 100% *M. oleifera* leaf extract, and 50% *A. indica* (neem) leaf extract - on postoperative pain and Substance P expression in teeth with symptomatic irreversible pulpitis and apical periodontitis. The findings demonstrated that all tested irrigants were associated with significant intragroup reductions in both postoperative pain and Substance P levels at 24 hours, indicating that final irrigation plays an important role in modulating inflammatory responses following single-visit endodontic treatment.

A significant reduction in Substance P levels was observed within each group from the preoperative to postoperative period. This finding is consistent with the established role of endodontic debridement and irrigation in reducing neurogenic inflammation by decreasing the microbial load and inflammatory mediators within the root canal system [5,6]. However, the magnitude of the reduction varied among the groups, as reflected by the differing effect sizes. Although neem and sodium hypochlorite demonstrated relatively larger effect sizes, ketorolac and *M. oleifera* showed smaller, but still statistically significant, reductions. These results suggest that, although all irrigants effectively reduced Substance P expression, the extent of neuropeptide modulation was not uniform across the groups.

Intergroup analysis revealed significant differences in baseline Substance P levels, indicating heterogeneity in preoperative inflammatory status among the study groups. This baseline variability likely reflects differences in individual inflammatory responses and disease severity, as previously reported in patients with symptomatic pulpitis and apical periodontitis [2-4]. Following instrumentation and irrigation, the intergroup differences in Substance P levels were markedly attenuated, with a substantially smaller effect size postoperatively. Post-hoc analysis showed that most baseline disparities were eliminated after treatment, except for a persistent difference between the ketorolac and neem groups. These findings indicate that endodontic intervention, irrespective of the irrigant used, contributes to the convergence of inflammatory mediator levels, highlighting the dominant role of canal disinfection and mechanical preparation in controlling pulpal inflammation.

Postoperative pain assessment using the VAS demonstrated a significant intragroup reduction in pain intensity in all four groups at 24 hours. This universal decrease is in agreement with previous reports, indicating that adequate chemomechanical preparation and obturation significantly reduce postoperative discomfort in teeth with irreversible pulpitis [2,3]. Importantly, no significant intergroup differences were observed in preoperative pain levels, confirming comparable baseline pain perceptions among the groups. However, significant differences emerged postoperatively, indicating that, while all irrigants reduced pain, their analgesic outcomes were not identical.

Post-hoc analysis revealed that the significant intergroup difference in postoperative pain was primarily attributable to higher pain scores in the neem group, whereas sodium hypochlorite, ketorolac, and *M. oleifera* showed comparable postoperative pain outcomes. The absence of significant differences among these three groups suggests that none demonstrated a clear statistical superiority in pain reduction. These findings align with earlier clinical studies reporting comparable analgesic outcomes between ketorolac and sodium hypochlorite-based irrigation protocols [16], as well as studies indicating that herbal irrigants may not consistently outperform conventional agents in pain control [17].

The anti-inflammatory and antioxidant properties of *M. oleifera*, attributed to its flavonoids and phenolic compounds, have been well documented in the pharmacological literature [10]. Similarly, the inhibition of cyclooxygenase-1 and prostaglandin synthesis by ketorolac supports its role in modulating inflammatory pain pathways [11,18]. However, in the present clinical context, these biological advantages did not translate into statistically superior postoperative pain or Substance P outcomes compared with sodium hypochlorite. This highlights the multifactorial nature of postoperative endodontic pain, where chemical modulation alone may be insufficient to override the effects of mechanical debridement, host responses, and procedural factors.

Sodium hypochlorite remains the most widely used irrigant because of its strong antimicrobial and tissue-dissolving properties [7,8]. Although higher concentrations have been associated with increased periapical irritation and postoperative pain [19-21], the present study did not demonstrate inferior pain outcomes with 5.25% sodium hypochlorite compared with ketorolac or *M. oleifera*. This suggests that, when used with controlled delivery and ultrasonic activation, higher concentrations may not necessarily exacerbate postoperative discomfort.

Neem demonstrated effective intragroup reductions in both pain and Substance P levels; however, its comparatively higher postoperative pain scores indicate a less consistent analgesic effect. While neem’s biocompatibility and endotoxin-reducing potential have been reported previously [9,17], its limited influence on the suppression of inflammatory mediators may account for the observed findings. Correlation analysis revealed weak and inconsistent relationships between Substance P levels and VAS pain scores across all groups, both preoperatively and postoperatively. This lack of a significant correlation suggests that postoperative pain perception cannot be solely attributed to changes in Substance P expression. These findings support the existing evidence that endodontic pain is influenced by multiple biological and psychosocial factors, including central sensitization, mechanical irritation, and individual pain thresholds [4,6].

Clinical implications

The findings of the present study suggest that effective chemomechanical preparation and final irrigation play critical roles in reducing postoperative pain and inflammatory mediator expression following single-visit endodontic treatment. All evaluated irrigants were associated with significant intragroup reductions in both pain intensity and Substance P levels at 24 hours. The comparable postoperative pain outcomes observed among sodium hypochlorite, ketorolac tromethamine, and *M. oleifera* indicate that alternative irrigants with anti-inflammatory or herbal origins may be considered in specific clinical situations, particularly where biocompatibility, patient preference, or sensitivity to conventional irrigants are concerns. Ketorolac tromethamine may be useful as an adjunct for inflammation-related pain modulation; however, its limited antimicrobial properties necessitate its use in conjunction with established disinfectants. Although *A. indica* demonstrated significant intragroup improvements, its comparatively higher postoperative pain scores suggest that its analgesic efficacy may be inconsistent. Overall, the results support the selective use of alternative irrigants as adjuncts rather than replacements for sodium hypochlorite, emphasizing the need for a balanced consideration of antimicrobial efficacy and biological response in clinical decision-making.

Limitations

Several limitations of the present study should be considered when interpreting the findings. First, the study employed a prospective, non-randomized observational design, and the irrigants were selected by the treating clinician rather than through random allocation. This introduces the potential for selection bias and unmeasured confounding factors, including differences in case complexity, operator preference, canal anatomy, and individual pain perception. Consequently, the results reflect associations rather than causal efficacy. Second, baseline samples were collected from the coronal inflamed pulp, whereas postoperative samples were obtained from the periapical region, and inherent biological differences between these tissues may have contributed to the observed reduction in Substance P levels, independent of the irrigant used. Third, postoperative pain was assessed at a single time point (24 hours), which may not capture delayed or persistent pain responses. In addition, pain assessment relied on patient-reported VAS scores, which are subjective and influenced by individual pain thresholds. Fourth, the herbal irrigants were prepared in-house, and although standardized protocols and fresh weekly preparation were followed, no physicochemical characterization (pH, osmolarity, or storage stability testing) was performed, and variability in phytochemical composition cannot be completely excluded. Finally, the sample size, while adequate for detecting differences in Substance P levels, limits the generalizability of the findings, and the study did not evaluate antimicrobial efficacy or long-term periapical healing. Future randomized controlled trials with standardized herbal formulations, uniform sampling strategies, extended follow-up, and comprehensive physicochemical and microbiological assessments are required to validate and expand upon these results.

## Conclusions

Within the limitations of this study, all tested irrigants - 5.25% sodium hypochlorite, ketorolac tromethamine, 100% *M. oleifera* leaf extract, and 50% *A. indica* leaf extract - were effective in significantly reducing postoperative pain and Substance P levels following single-visit endodontic treatment of teeth with symptomatic irreversible pulpitis and apical periodontitis. Intergroup differences in postoperative pain were primarily influenced by less consistent analgesic outcomes with *A. indica*, while sodium hypochlorite, ketorolac, and *M. oleifera* demonstrated comparable clinical performance. These findings support the adjunctive use of alternative irrigants, emphasizing the importance of balancing antimicrobial efficacy with biological response.
